# Atomic scale displacements detected by optical image cross-correlation analysis and 3D printed marker arrays

**DOI:** 10.1038/s41598-021-81712-8

**Published:** 2021-01-27

**Authors:** Tobias Frenzel, Julian Köpfler, Andreas Naber, Martin Wegener

**Affiliations:** 1 Institute of Applied Physics, Karlsruhe Institute of Technology (KIT), 76128 Karlsruhe, Germany; 2 Institute of Nanotechnology, Karlsruhe Institute of Technology (KIT), 76021 Karlsruhe, Germany

**Keywords:** Applied optics, Optical techniques, Imaging and sensing

## Abstract

For analyzing displacement-vector fields in mechanics, for example to characterize the properties of 3D printed mechanical metamaterials, routine high-precision position measurements are indispensable. For this purpose, nanometer-scale localization errors have been achieved by wide-field optical-image cross-correlation analysis. Here, we bring this approach to atomic-scale accuracy by combining it with well-defined 3D printed marker arrays. By using an air-lens with a numerical aperture of $0.4$ and a free working distance of $11.2\, \mathrm{mm}$, and an $8\times 8$ array of markers with a diameter of $2\, \upmu\mathrm{m}$ and a period of $5\,\upmu \mathrm{ m}$, we obtain 2D localization errors as small as $0.9\, \AA$ in $12.5\, \mathrm{ms}$ measurement time ($80\, \mathrm{frames}/\mathrm{s}$). The underlying experimental setup is simple, reliable, and inexpensive, and the marker arrays can easily be integrated onto and into complex architectures during their 3D printing process.

## Introduction

Highly accurate position and displacement measurements are of tremendous importance in many applications, ranging from the detection of gravitational waves to industrial metrology to materials characterization in mechanics. The laws of classical physics do not impose any fundamental limits on the accuracy with which one can measure the position of an object. In quantum mechanics^1^, the standard deviation of the position measurement value, $s$, is subject to fundamental quantum mechanical uncertainty; however, the standard error of the mean or localization error, $\sigma$, can approach zero—as in classical physics. Only statistics limits the achievable accuracy. Therefore, in general, a goal of position and displacement metrology is to achieve a given $\sigma$ in as short a time as possible or to obtain minimum $\sigma$ in a given time. This optimization must appreciate constraints that may apply depending on the application. For example, mechanical contact to the sample may not be acceptable, in which case optical approaches are attractive. Furthermore, fluorescence detection may or may not be possible, a minimum physical distance to the sample could be required, etc.

Optical approaches aiming at determining position vectors with ultra-small localization errors include laser interferometry^2–6^, laser Doppler vibrometry^7–9^, fluorescence-based single-molecule localization^10–13^, light-scattering-based single-particle localization^14^, localization by optical superoscillations from metasurfaces^15^, and optical-image cross-correlation analysis^16–21^.

Concerning acoustical or mechanical metamaterials, laser Doppler vibrometry has frequently been used for measuring the out-of-plane displacement-vector component^22,23^. Sub-picometer precision is routinely available by commercial instruments^24^. Image cross-correlation analysis has widely been used for measuring the in-plane components. Here, nanometer-precision characterization of mechanical metamaterials has been achieved. For all of these applications, contact-free measurements at centimeter-scale working distances or beyond, without the need for fluorescent labels, are absolutely crucial^19,20^. However, some of the optical-image cross-correlation experiments were performed close to the noise limit defined by the accessible localization errors^21,25^. Therefore, smaller localization errors would have been highly desirable.

The novelty of this paper is to push the optical-image cross-correlation approach towards atomic-scale localization errors, while maintaining all of its other virtues. As pointed out above, only statistics limits the achievable accuracy. For certain sample surfaces and under special fortuitous conditions, the statistics can be improved by using multiple regions of interest. However, to make the approach reliable, robust, and versatile, we introduce 3D printed 2D arrays of small and well-defined optical markers. Using an $8\times 8$ array of markers within a $(40\,\upmu {\mathrm{m})}^{2}$ measurement footprint, we obtain a mean localization error of less than one Angstrom within $12.5\ \mathrm{ms}$ measurement time, equivalent to $80\, \mathrm{frames}/\mathrm{s}$ frame rate.

## Methods

### Optical-image cross-correlation analysis

Optical-image cross-correlation analysis^16^ starts with two optical images of the same object, ${I}_{1} (x,y)$ and ${I}_{2}(x,y)$, in the $xy$-image plane. These signals can, for example, be derived from an optical bright-field microscope connected to a digital camera, in which case $x={n}_{x}p$ and $y={n}_{y}p$ are pixelated, with pixel size $p$ and integers ${n}_{x}$ and ${n}_{y}$. Unlike for the single-particle tracking approaches cited above, the images need not necessarily be taken at the ultimate diffraction limit. In other words: It is possible to use low numerical-aperture microscope lenses. The images will generally contain perturbations, e.g., shot noise, excess electrical read-out noise, stray light, or combinations thereof. To derive a possible nonzero displacement vector, $(\delta x,\delta y)$, between the two images #1 and #2, we first calculate the two-dimensional (2D) cross-correlation function

1 $$C\left(\Delta x,\Delta y\right)=\int {I}_{1}\left(x,y\right){I}_{2}\left(x+\Delta x,y+\Delta y\right){\mathrm{d}}x {\mathrm{d}}y.$$

This integral can be performed over the entire available image or over only selected small regions of it, which we refer to as the regions of interest (ROI). This selection is based on large-contrast fine features within the ROI. We select $M$ different ROI, corresponding to $M$ individual measurements, from which we later compute the mean value and the localization error (see below). This procedure is justified if the systematic error due to the relative motion between these ROI during one measurement is smaller than the determined localization error. For a pixelated image, the integral in Eq. (1) reduces to a sum and the displacement components, $\Delta x$ and $\Delta y$, are integer multiples of the pixel size $\left(\Delta x,\Delta y\right)=\left(\Delta {n}_{x}p,\Delta {n}_{y}p\right).$ Provided that the shift of the object between the two images ${I}_{1}$ and ${I}_{2}$ is much smaller than the pixel size in the object plane, the cross-correlation function will exhibit a single maximum at $(\Delta x,\Delta y)=(\mathrm{0,0})$, possibly with noise on top. For each ROI, we determine the displacement vector with subpixel precision $(\delta x,\delta y)$ by a least-squares fit of a two-dimensional parabola to the maximum of $C(\Delta x,\Delta y)$ over $3\times 3$ pixels (each ROI corresponds to $30\times 30$ pixels). This overall procedure is implemented in an open-access software package^26^, which we have used for the image analysis in this paper. It has previously been used by us^19–21,25^. Here, we have also tested the software by feeding it with computer generated images ${I}_{1}$ and ${I}_{2}$ corresponding to a displacement of, e.g., $1.0\,\mathrm{nm}$, leading to a retrieved displacement of $1.0\, \mathrm{nm}$ indeed (not depicted). Further simulations are described below.

### Localization errors

We use the common definitions of the standard deviation $s$, and the standard error of the mean or localization error $\sigma$. For all quantities, we distinguish between the $x$- and the $y$-component by corresponding indices.

For $M\gg 1$ (with $M$ ROI as defined above) individual measurements at one position, we compute the standard deviation ${s}_{x}$ as

2 $${s}_{x}=\sqrt{\sum_{i=1}^{M}\frac{{\left(\delta {x}_{i}-\langle \delta x\rangle \right)}^{2}}{M-1} .}$$

Here, $\langle \delta x\rangle =\left(\sum_{i=1}^{M}\delta {x}_{i}\right)/M$ is the mean value. This procedure is meaningful if the variances of the position determination for the $M$ ROI are similar. This aspect has been verified for the data to be shown below. The localization error is given by

3 $${\sigma }_{x}=\frac{{s}_{x}}{\sqrt{M}} .$$

The quantities ${s}_{y}$ and ${\sigma }_{y}$ are defined analogously.

### Setup

**Figure 1:**
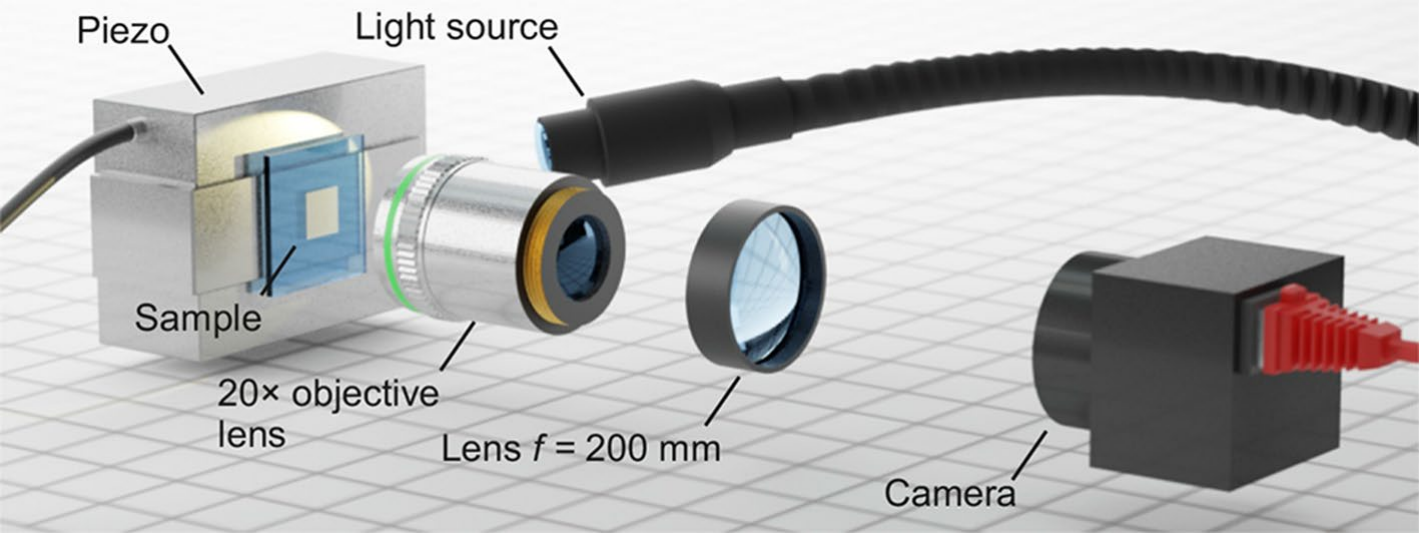
Scheme of the simple optical setup used to determine two-dimensional displacement vectors of a macroscopic sample with atomic-scale localization errors. The surface of a sample is illuminated by unpolarized visible white light from a filtered incandescent source impinging onto the sample under an angle. An objective lens (with focal length $f=8.25\, \mathrm{mm}$) together with a tube lens (with focal length $f=200\, \mathrm{mm}$) images the sample surface onto a digital black/white camera. The objective lens has a numerical aperture of $\mathrm{NA}=0.4$ and a free working distance of $11.2\,\mathrm{ mm}$. The images acquired by the camera are processed using image cross-correlation analysis. We can displace the sample in the plane normal to the optical axis by a precision piezoelectric stage. The setup is located on a vibration-isolated optical table and enclosed in a box to reduce vibrations and drifts between the sample and the camera position.

Our simple home-built microscope setup shown in Fig. 1 is composed of one microscope objective lens (Zeiss LD Achroplan 20 × /0.40 Corr., $\mathrm{NA}=0.4$, free working distance $11.2\,\mathrm{mm}$) and one tube lens (Thorlabs SC254-200-A-ML, focal length $200\, \mathrm{mm}$). This microscope images the sample plane onto a silicon complementary metal–oxide–semiconductor (CMOS) black/white camera chip (Sony IMX264, $2448\times 2048\,\mathrm{ pixels}$), which is connected to a computer. One pixel of the camera chip in the image plane has a side length corresponding to $138.6\,\mathrm{ nm}$ in the sample plane. We operate the camera at its maximum frame rate of $80\, \mathrm{frames}/\mathrm{s}$ $=1/(12.5\, \mathrm{ms})$, corresponding to an individual exposure time of $12.26\, \mathrm{ms}$ plus a read-out time of about $0.24\, \mathrm{ms}$. This frame rate requires reading out only $512\times 512\,\mathrm{ pixels}$ of the camera chip. $512$ pixels correspond to a length of about $71 \,\upmu\mathrm{m}$. This length is much smaller than the diameter of the field of view of about $1\, \mathrm{mm}$ (in the sample plane). Therefore, we assume that image distortions are negligible for the investigated area. We illuminate the sample by a standard swan-neck incandescent lamp (Schott KL 1500 LCD, with additional Thorlabs FESH0700 cold filter) emitting visible white light, which is directed onto the sample under an angle with respect to the optical axis (see Fig. 1). This illumination is sufficiently bright to take full advantage of the camera’s dynamic range of $8\, \mathrm{bit}$ within the exposure time of $12.26\,\mathrm{ ms}$ (see below), while not overloading it. The sample can be translated by a precision one-axis piezoelectric translation stage (Physik Instrumente P-753.1CD) with capacitive position read-out and the possibility of active feedback control (Physik Instrumente digital controller E-710.3CD). This stage is specified with a resolution of $0.1\, \mathrm{nm}$.

To quantify the contribution of the read-out noise, we also define the standard error of the mean ${\sigma }_{x}^{^{\prime}}$ for the nominal $x$-position obtained by the capacitive sensor. For each frame, we average over $K=5$ sensor measurements acquired within the corresponding exposure time, such that

4 $${\sigma }_{x}^{^{\prime}}=\sqrt{\sum_{k=1}^{K}\frac{{\left(\delta {\stackrel{\sim}{x}}_{k}-\langle \delta {\stackrel{\sim}{x}}\rangle \right)}^{2}}{K(K-1)} .}$$

Here, $\langle \delta{ \stackrel{\sim }{x}}\rangle =\left(\sum_{k=1}^{K}\delta {\stackrel{\sim }{x}}_{k}\right)/K$ is the corresponding mean value.

## Results

### Experimental results

**Figure 2:**
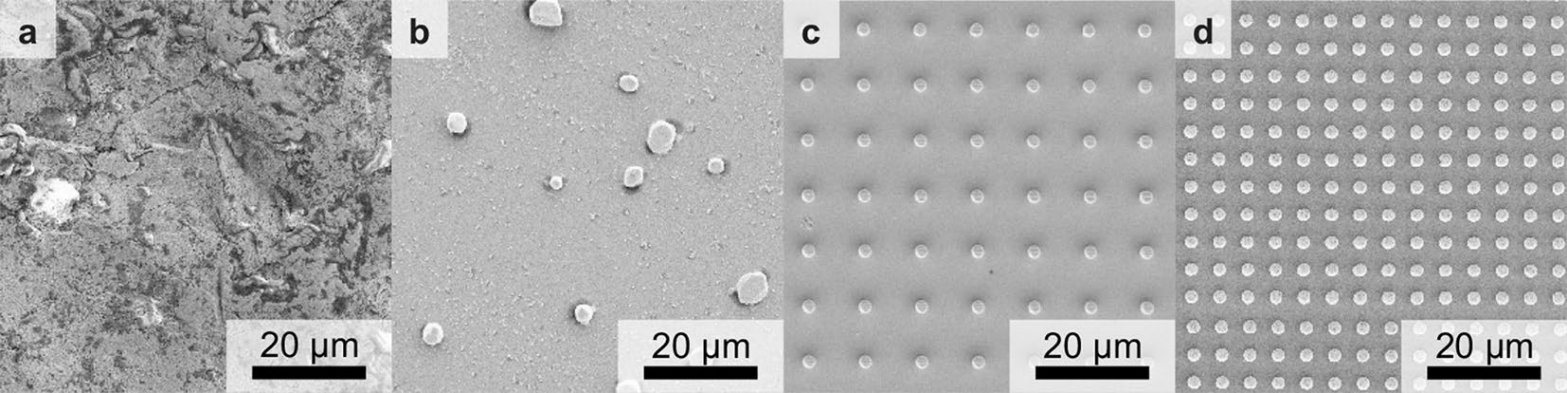
Top-view electron micrographs of four of the five investigated samples. (**a**) Sample #1 is a sandblasted copper surface. (**b**) Sample #2 is a glass substrate with randomly distributed micrometer-sized gold grains on top. Sample #3 (not depicted) is a glass substrate with a square array of polymer markers with period $a=10\,\upmu \mathrm{m}$ on top, fabricated by 3D laser printing. Without metal coating, this sample cannot easily be imaged by electron microscopy. (**c**) Sample #4 is as sample #3, but coated with a $54\,\mathrm{ nm}$ thin film of gold. (**d**) Sample #5 is as sample #4, but with a period of $a=5\, \upmu \mathrm{m}$.

**Figure 3:**
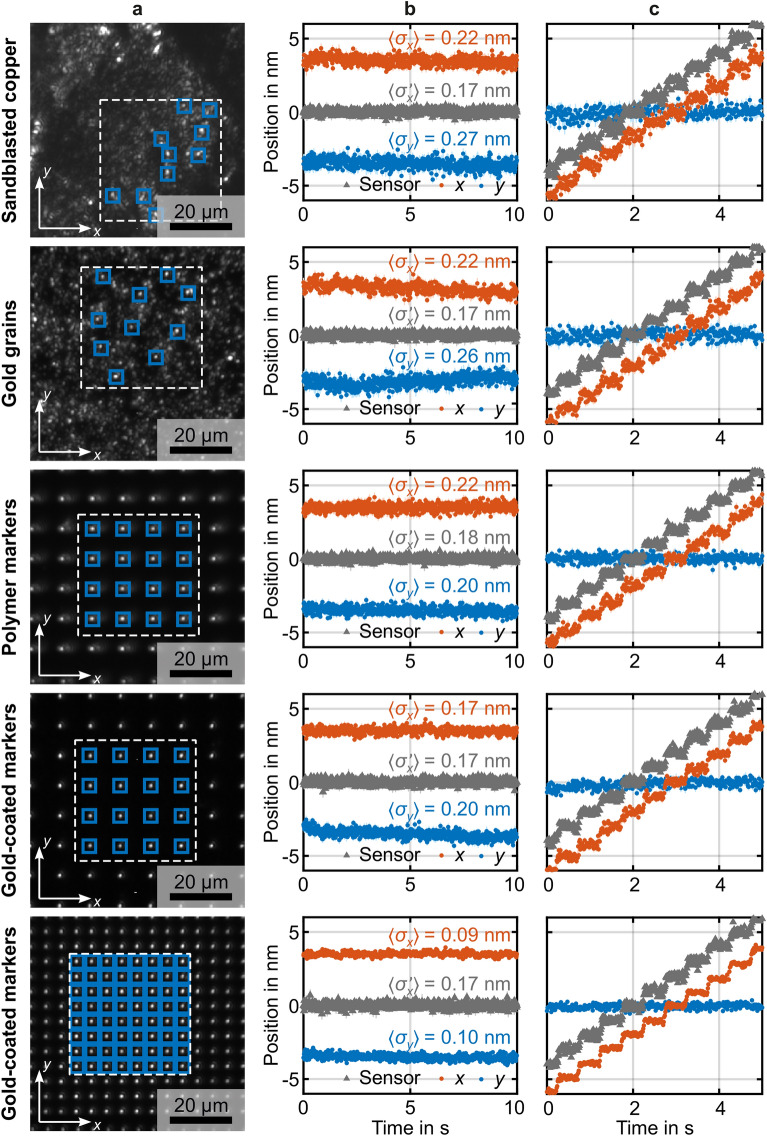
Summary of data obtained from five different samples #1 to #5 (cf. Fig. 2). Column (**a**) exhibits an example optical image with the used regions of interest (ROI) indicated by the blue squares. Each ROI comprises $30\times 30$ camera pixels. The ROI lie in a footprint of ${\left(40\, \upmu\mathrm{m}\right)}^{2}$ indicated by the dashed white square. Column (**b**) shows results obtained from the optical-image cross-correlation approach for the $x$-component (red) and the $y$-component (blue). For comparison, the read-out signal from the capacitive sensor of the piezoelectric actuator is shown in gray. This signal has been shifted vertically for clarity. For each of the $800$ data points, we obtain localization errors ${\sigma }_{x}$ and ${\sigma }_{y}$. The mean values $\langle {\sigma }_{x}\rangle$ and $\langle {\sigma }_{x}\rangle$ over $800$ measurements are indicated. $\langle {\sigma }_{x}^{^{\prime}}\rangle$ is the corresponding value for the capacitive sensor, for the same measurement time of $12.5\, \mathrm{ms}$. In column (**b**), the piezoelectric actuator has not been moved intentionally. In contrast, in column (**c**), the piezoelectric actuator has been moved in a staircase manner with $1\,\mathrm{nm}$ high steps each $0.5\, \mathrm{s}$.

We illustrate the optical-image cross-correlation approach using a set of different samples. Four electron micrographs are shown in Fig. 2. Sample #1 depicted in Fig. 2a is a sandblasted copper surface. The optical image of sample #1 exhibited in the first row of Fig. 3 is partly due to interference effects, which give rise to spatially narrow and high-contrast features. In Fig. 3, we will show a best-case example. However, typical examples are much worse. Sample #2 depicted in panel **b** consists of micrometer-sized gold grains that are randomly distributed on an optical-quality glass surface. The gold grains offer an easy way to provide high-contrast features to arbitrary low-contrast structures. However, the disordered arrangement of the grains makes the results very much dependent on the chosen sample position. In Fig. 3, we will again display a best-case example. Figure 2c shows a glass surface onto which we have added a periodic square array of polymer markers with a diameter of about $d=2 \, \upmu\mathrm{m}$ and a period of $a=10\,\upmu\mathrm{m}$. We have manufactured these markers by using standard 3D laser lithography^27^, using the commercial system Photonic Professional GT with photoresist IP-Dip (both Nanoscribe GmbH, Germany) and a 63x/1.4 NA objective. Thereafter, we have sputtered a $54\,\mathrm{ nm}$ thin film of gold onto this sample #4. Sample #3 is as sample #4, but without the sputtered gold film. Without a conductive layer, this sample cannot easily be imaged by electron microscopy. Sample #5, which is depicted in Fig. 2d, is as sample #4 but for a period of $a=5\, \upmu\mathrm{ m}$.

Results for samples #1 to #5 are summarized in Fig. 3. The five samples correspond to the five rows of this $5\times 3$ matrix. The three columns **a**-**c** exhibit different measurements. The panels in **a** show typical raw camera images that are fed into the optical-image cross-correlation analysis. The $M$ ROI used for the analysis are indicated by the blue squares. They contain $30\times 30$ camera pixels each for all samples. Note that $M$ varies among the samples as indicated. All used ROI lie in an area in the image plane corresponding to a footprint of ${\left(40\,\upmu\mathrm{m}\right)}^{2}$ in the sample plane (dashed white square). The panels in column **b** show the $x$- and the $y$-component of the displacement vector, and the nominal $x$-position of the 1D capacitive sensor, for $800$ points in time corresponding to a total time of $10 s$. For each of these $800$ points, the colored error bars correspond to $\pm 1{\sigma }_{x},$ $\pm 1{\sigma }_{y}$, and $\pm 1{\sigma }_{x}^{^{\prime}}$, respectively. To a large extent, the error bars are smaller than the symbol size. The mean values of ${\sigma }_{x}$, ${\sigma }_{y}$, and ${\sigma }_{x}^{^{\prime}}$ for the $800$ points for the $x$- and the $y$-component, $\langle {\sigma }_{x}\rangle$, $\langle {\sigma }_{y}\rangle$, and $\langle {\sigma }_{x}^{^{\prime}}\rangle$, are indicated. Here, the sample has not been moved intentionally. Both the $x$- and $y$-components exhibit typical drifts which are due to a relative motion between sample and camera. The drifts tend to be yet larger if we remove the housing covering the setup (not depicted). Without the housing, unwanted displacements can be induced by airflow, increased temperature variations, and by external sound sources. The panels in column **c** exhibit the same quantities as in panels **b**, however, we now intentionally move the piezoelectric stage in a staircase manner with a step height of $1\ \mathrm{nm}$. In column **c**, for all samples, the steps in the $x$-direction can be seen clearly, in addition to the slower and subtle drift motions. This observation provides a first and intuitive confirmation that the localization error achieved by the optical-image cross-correlation approach is much less than one nanometer indeed.

As we obtain a localization error for each image, corresponding to one data point in Fig. 3b, it is not meaningful to quote all localization errors individually. We rather quote for each sample the average value, $\langle {\sigma }_{x}\rangle$, over $800$ camera images. Inspecting rows 4 and 5 of Fig. 3, one can clearly see that the localization error decreases with increasing number $M$ of markers in the array. Furthermore, from row 3 to row 4, the localization error decreases when improving the image quality and image contrast by going from the bare polymer dots to the gold-coated polymer dots.

The localizations errors shown in rows 1 and 2 are respectable, too. However, it must be noted that the depicted data are best-case examples taken on sample positions where we have fortuitously found a large number of well-localized and high-contrast bright spots. For many other sample positions (not depicted), we have found much worse results for the sand-blasted copper surface and for the surface covered with gold grains, respectively. Therefore, these approaches do not reliably provide sub-nanometer localization errors. In sharp contrast, the small localization errors on the samples including 3D printed marker arrays are immediately reproducible after a setup realignment and, hence, reliable. For example, the experiments in row 5 of Fig. 3 have been repeated 5 times. We find the same localization error within $\pm 1.5\%$ (not depicted).

### Simulation of localization errors

References^16^ and^18^ give an overview of the various statistical and systematic errors in digital image cross-correlation analysis. In particular, the combination of finite pixel size and finite number of bits already has a significant contribution to the measured localization error^28,29^. To explore the limits for the localization error for our specific conditions, we have performed computer simulations in which we have generated $8\times 8$ arrays of Gaussian light spots with a width and arrangement comparable to those of sample #5 (see panel **a** in the fifth row of Fig. 3). Furthermore, we have considered the same pixel numbers for the ROI and for the fitting as well as a number of $8$ bits as in the experiments (see above). In our simulations, each pixel averages over the intensity within. The brightness of the light spots was chosen to cover the full $8$ bit dynamic range of the image. The processing of the simulated data was strictly identical to that of the experimental data. Accounting for read-out noise with an amplitude of, e.g., $2.3$ bits for each camera pixel has led to simulated statistical localization errors of $\langle {\sigma }_{x}^{\mathrm{sim}}\rangle =0.08\,\mathrm{ nm}$ and $\langle {\sigma }_{y}^{\mathrm{sim}}\rangle =0.08\,\mathrm{ nm}$ (not depicted). These values are comparable to ${\langle \sigma }_{x}\rangle =0.09\, \mathrm{nm}$ and ${\langle \sigma }_{y}\rangle =0.10\, \mathrm{nm}$ obtained for sample #5 (see panel **b** in the fifth row of Fig. 3). To investigate systematic errors^28,29^, we have located the spots at various different positions with respect to the simulated camera pixel array. Thereby, for zero read-out noise, we have obtained simulated localization errors of $\langle {\sigma }_{x}^{\mathrm{sim}}\rangle =0.02\, \mathrm{ nm}$ and $\langle {\sigma }_{y}^{\mathrm{sim}}\rangle =0.03\,\mathrm{ nm}$. These simulated systematic errors show that the localization errors achieved in our experiments already approach the limit of the underlying image cross-correlation algorithm under the given conditions.

Finally, Figure S1 illustrates the dependence of the localization error on the width of the light spots and their brightness. As expected, the localization error increases with increasing spot width and decreasing brightness level.

## Discussion

The image cross-correlation approach as presented here allows for determining the two in-plane components of the displacement-vector field with small localization errors simultaneously, for a total area for the used markers of ${\left(40\, \upmu \mathrm{m}\right)}^{2}$, and for a large free working distance of the sample to the microscope lens of $11.2\,\mathrm{ mm}$. For example, for the $8\times 8$ gold-coated polymer-marker array presented in the last row of Fig. 3, we have achieved a mean localization error of ${\langle \sigma }_{x}\rangle =0.09\, \mathrm{nm}$ at $12.5\, \mathrm{ms}$ time resolution. This value is significantly better than anything else that we have previously obtained by using the image cross-correlation approach on samples without dedicated marker arrays. This finding correlates positively with the fact that this sample has the largest density of non-overlapping ROI in the given footprint of ${\left(40\, \upmu \mathrm{m}\right)}^{2}$. The image acquisition process itself is identical to not using marker arrays. The fabrication of the marker arrays onto 3D printed mechanical metamaterial architectures takes negligible time compared to that of the rest of such samples.

For the same measurement time of $12.5\, \mathrm{ms}$, equivalent to a camera frame rate of $80$ $\mathrm{frames}$/s, the localization error obtained from the $8\times 8$ gold-coated marker-array sample in the last row of Fig. 3 is even significantly smaller than the localization error $\langle {\sigma }_{x}^{^{\prime}}\rangle =0.17\, \mathrm{nm}$ obtained from the capacitive sensor that is built into the high-quality piezoelectric actuator. However, to be fair, it should be noted that the signal obtained from the capacitive sensor is available in real time. The data for the optical-image cross-correlation approach are also acquired in real time, but the subsequent cross-correlation analysis described above takes considerable overhead time. On a state-of-the-art standard personal computer, it has taken us about $15\ \mathrm{ms}$ processing time for one ROI in one image, of which $7\, \mathrm{ms}$ are required to merely load the image into the software. With an increasing number of markers, the software overhead per marker decreases. For example, for $8\times 8$ ROI, this leads to a processing time of $90\ \mathrm{ms}$ for one image and to $72\, \mathrm{s}$ processing time total for the $800$ images for each sample shown in Fig. 3b. This timescale is essentially irrelevant when performing high-precision characterization experiments on mechanical metamaterials, which is the application we have in mind. However, if one aims at any sort of real-time active feedback of a displacement or position, this timescale would obviously be unacceptable. It has been shown for cross-correlation analysis that the processing time can be sped up substantially by using field-programmable gate arrays (FPGA)^30,31^ instead of the single standard personal computer in our experiments.

Finally, we note that adding markers to a sample generally influences its properties. For example, the (metallized) polymer markers may influence the local dielectric properties and will increase the optical scattering. Likewise, the additional mass will affect the mechanical properties of the specimen under investigation. However, in previous experiments comparable markers have not had a major disturbing influence, neither in quasi-static regime^32^, nor in measurements at ultrasound frequencies^25^.

## Conclusion

By introducing well-defined 3D printed marker arrays on surfaces, we have reliably pushed the optical-image cross-correlation approach to localization errors below one Angstrom at camera frames rates of $80\, \mathrm{frames}/\mathrm{s}$. Under our conditions, one Angstrom is several thousand times smaller than the wavelength of white light used for illumination and more than thousand times smaller than a single camera pixel. Most importantly, these values are achieved with a very simple and inexpensive optical setup that can immediately be used for applications, e.g., for the characterization of mechanical metamaterials.

## Supplementary Information

Supplementary Figure 1.

## Data Availability

The datasets generated during and/or analysed during will be provided upon reasonable request and are published in the open repository KITopen.
