# Supplementary material for: Atomic scale displacements detected by optical image cross-correlation analysis and 3D printed marker arrays
Source: Sci Rep. 2021 Jan 27;11:2304. doi: 10.1038/s41598-021-81712-8 (PMC7840920; doi:10.1038/s41598-021-81712-8)
Supplement: Supplementary file 1 — Supplementary Figure 1. [file 41598_2021_81712_MOESM1_ESM.pdf]

Supplementary Information

Atomic scale displacements detected by optical image cross-correlation analysis and 3D printed marker arrays

Tobias Frenzel[1,†], Julian Köpfler[1,2,†], Andreas Naber[1], and Martin Wegener[1,2,*]

[†]*these authors have contributed equally to this work*

[1]*Institute of Applied Physics, Karlsruhe Institute of Technology (KIT), 76128 Karlsruhe, Germany*

[2]*Institute of Nanotechnology, Karlsruhe Institute of Technology (KIT), 76021 Karlsruhe, Germany*

**martin.wegener@kit.edu*

Supplementary Figure S1

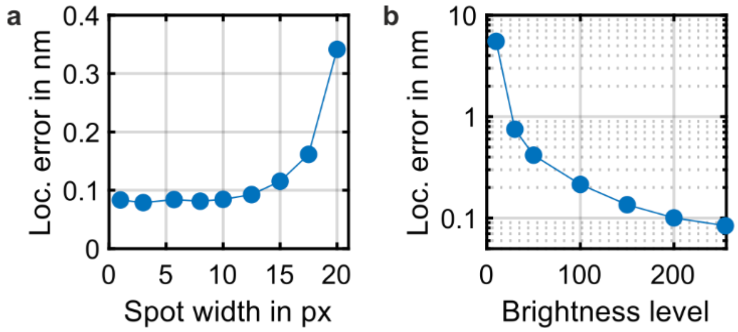

Fig. S1. Simulated localization error versus light-spot width and brightness level. (**a**) Dependence as a function of the width of the light spot. (**b**) Dependence as a function of spot brightness. The used parameters are a read-out noise with an amplitude of 2.3 bits and a pixel scale of 138.6 nm/px.
